# Therapeutic Potential of Heterocyclic Compounds Targeting Mitochondrial Calcium Homeostasis and Signaling in Alzheimer’s Disease and Parkinson’s Disease

**DOI:** 10.3390/antiox12061282

**Published:** 2023-06-15

**Authors:** Victor Tapias, Paula González-Andrés, Laura F. Peña, Asunción Barbero, Lucía Núñez, Carlos Villalobos

**Affiliations:** 1 Unidad de Excelencia Instituto de Biomedicina y Genética Molecular de Valladolid (IBGM), Universidad de Valladolid y Consejo Superior de Investigaciones Científicas (CSIC), 47003 Valladolid, Spain; 2 Departamento de Bioquímica y Biología Molecular y Fisiología, Facultad de Medicina, Universidad de Valladolid, 47003 Valladolid, Spain; 3 Departamento de Química Orgánica, Facultad de Ciencias, Universidad de Valladolid, 47003 Valladolid, Spain

**Keywords:** Alzheimer’s disease, Parkinson’s disease, mitochondria, oxidative stress, calcium, heterocyclic compounds

## Abstract

Alzheimer’s disease (AD) and Parkinson’s disease (PD) are the two most common neurodegenerative diseases in the elderly. The key histopathological features of these diseases are the presence of abnormal protein aggregates and the progressive and irreversible loss of neurons in specific brain regions. The exact mechanisms underlying the etiopathogenesis of AD or PD remain unknown, but there is extensive evidence indicating that excessive generation of reactive oxygen species (ROS) and reactive nitrogen species (RNS), along with a depleted antioxidant system, mitochondrial dysfunction, and intracellular Ca^2+^ dyshomeostasis, plays a vital role in the pathophysiology of these neurological disorders. Due to an improvement in life expectancy, the incidence of age-related neurodegenerative diseases has significantly increased. However, there is no effective protective treatment or therapy available but rather only very limited palliative treatment. Therefore, there is an urgent need for the development of preventive strategies and disease-modifying therapies to treat AD/PD. Because dysregulated Ca^2+^ metabolism drives oxidative damage and neuropathology in these diseases, the identification or development of compounds capable of restoring Ca^2+^ homeostasis and signaling may provide a neuroprotective avenue for the treatment of neurodegenerative diseases. In addition, a set of strategies to control mitochondrial Ca^2+^ homeostasis and signaling has been reported, including decreased Ca^2+^ uptake through voltage-operated Ca^2+^ channels (VOCCs). In this article, we review the modulatory effects of several heterocyclic compounds on Ca^2+^ homeostasis and trafficking, as well as their ability to regulate compromised mitochondrial function and associated free-radical production during the onset and progression of AD or PD. This comprehensive review also describes the chemical synthesis of the heterocycles and summarizes the clinical trial outcomes.

## 1. Introduction

The brain is particularly prone to oxidative insult due to a complex interconnected myriad of factors, such as high metabolic activity, neurotransmitter autoxidation, elevated content of redox active transition metals, modest antioxidant defense, glutamate excitotoxicity, and altered calcium (Ca^2+^) influx and associated signaling processes. An impaired antioxidant system or aberrant and sustained free-radical formation can result in redox balance modifications and concomitant alteration in redox-sensitive signaling pathways. At the physiological level, brain reactive oxygen species (ROS) and reactive nitrogen species (RNS) are second messengers involved in intracellular signaling, but increased levels of free radicals cause harmful effects to biological macromolecules that contribute to the aging process and the pathogenesis of neurodegenerative diseases. Lipid peroxidation (LPO) consists of the abstraction of allylic hydrogen atoms from side-chains of polyunsaturated fatty acids (PUFAs) by ROS and RNS. A major deleterious outcome of LPO is the production of a variety of reactive aldehyde species, such as malondialdehyde (MDA) and 4-hydroxy-2-nonenal (4-HNE). The reaction between O_2_^•−^ and NO^•^ leads to the formation of peroxynitrite, which targets tyrosine residues in proteins via free-radical addition to generate 3-nitrotyrosine (3-NT). Protein carbonylation is an oxidative stress-driven nonenzymatic and irreversible post-translational modification (PTM). Oxidative deamination of alkaline amino acids such as arginine, lysine, and histidine leads to protein carbonylation. Advanced glycation end products (AGEs) are generated in a nonenzymatic reaction involving lipids, proteins, or nucleic acids and reducing sugars. The interaction of AGEs with their receptors (RAGEs) causes oxidative stress. ROS/RNS may also interact with nucleobases of the DNA (e.g., guanine) to form 8-hydroxy-2-deoxyguanosine (8-OHdG) while oxidative RNA damage produces 8-hydroxyguanosine (8-OHG).

Compelling evidence has demonstrated that the mitochondrial electron transport chain (ETC) is the major endogenous source of ROS/RNS generation, although the endoplasmic reticulum (ER) and peroxisomes can also be an important cause of free-radical production [1]. Specifically, respiratory chain complexes I (NADH: ubiquinone oxidoreductase) and III (ubiquinol: cytochrome c oxidoreductase) generate high rates of O_2_^•−^. The ETC also comprises membrane-embedded proteins in the inner mitochondrial membrane that shuttle electrons from NADH and FADH_2_ to molecular oxygen. Simultaneously, protons are pumped from the mitochondrial matrix to the intermembrane space, thereby resulting in the reduction of oxygen to water. The energy released from these redox reactions is stored as a mitochondrial potential used to drive the phosphorylation of adenosine diphosphate (ADP) to form adenosine triphosphate (ATP). Moreover, PTMs involve enzyme-mediated covalent addition of specific functional groups (such as phosphorylation, ubiquitination, and nitration) to proteins after their synthesis. Redox-related PTMs can modulate the activity of proteins implicated in a variety of cellular signaling pathways, such as protein folding and degradation, transcription factor expression and activity, and energy metabolism by regulating the tricarboxylic acid cycle (TCA) and glycolytic enzymes, fatty-acid metabolism, and protein cysteine thiol nitrosation, sulfenylation, or glutathionylation. Mitochondria along with the ER play an important role in controlling intracellular Ca^2+^ homeostasis, which regulates several neuronal processes, including synaptic plasticity, metabolic regulation, proliferation, gene expression, and apoptosis.

Alzheimer’s disease (AD) and Parkinson’s disease (PD) are two neurodegenerative diseases that share common pathological features, including protein misfolding and aggregation, synaptic impairment, mitochondrial deficits, axonopathy, and aberrant free-radical production. Ca^2+^ can behave as a pathological effector in these neurological disorders. Cytosolic and organelle Ca^2+^ overload promotes its mitochondrial accumulation, which triggers the opening of the mitochondrial permeability transition pore (mPTP) and the disruption of the mitochondrial membrane potential (ΔΨm). Sustained mPTP opening leads to Ca^2+^ release, mitochondrial depolarization, OXPHOS disruption, compromised structural and functional integrity of the inner mitochondrial membrane, and release of cytochrome c (CYT C) and other apoptotic proteins from the outer mitochondrial membrane [2]. Ca^2+^ overload is mainly mediated by Aβ and tau in AD and α-synuclein (α-syn) and leucine-rich repeat kinase 2 (LRRK2) in PD. Modifications in neuronal Ca^2+^ influx via voltage-gated Ca^2+^ channels (VOCCs) and glutamate receptors promote excitotoxic Ca^2+^ accumulation and concomitant defective neurotransmission, impaired synaptic plasticity, damaged mitochondrial function, and increased ROS/RNS production. This review illustrates the potential therapeutic value of several heterocyclic compounds that modulate Ca^2+^ signaling and homeostasis and may be used as therapeutic agents for the treatment of neurodegenerative diseases.

## 2. Mitochondrial Deficiencies and Oxidative Stress as Close Partners in Alzheimer’s Disease

AD is the most common neurodegenerative disorder characterized by brain atrophy and impaired cognitive performance. Neuropathological studies have observed an extracellular accumulation of amyloid beta (Aβ) and an intraneuronal deposition of insoluble neurofibrillary tangles (NFTs) containing hyperphosphorylated tau protein. A large body of research has shown that impaired mitochondrial function (and related energy failure) is an early causative factor of AD. Brain metabolic variations would be primarily responsible for mitochondrial dysfunction in AD. Glucose deprivation leads to reduced activity in the default mode network, an area associated with atrophy and amyloid and tau deposition in AD. Positron emission tomography (PET) with [^18^F]-fluro-2-deoxyglucose demonstrated a progressive decline in cerebral glucose metabolism in AD patients [3]. There is a direct relationship between reduced brain regional glucose metabolism and downregulated thiamine-dependent enzyme activities, such as α-ketoglutarate dehydrogenase complex (α-KGDH) and the pyruvate dehydrogenase complex (PDHC). However, the most common feature observed in AD is a deficiency in mitochondrial complex IV (CYT C oxidase, COX), which has been reported in the cortical and hippocampal brain regions and platelets of patients [4,5]. Another specific event linked to mitochondrial failure in AD is the development of phenotypic changes in these organelles. Mitochondrial morphometric abnormalities and reduced numbers of intact mitochondria were found in AD brain specimens [6]. Mitochondrial dynamics serve as a quality control mechanism and are tightly regulated by the fusion-fission machinery, which promotes the generation or degradation of a mitochondrial syncytium. Axonal transport is a cellular mechanism that controls the active trafficking of proteins, lipids, organelles, and neurotransmitters. Mitochondrial axonal transport is tightly interconnected with mitochondrial dynamics. mRNA and protein levels of Opa1, Mfn1, and Mf2 were reduced while those of Drp1 and Fis1 were upregulated in postmortem brain samples from individuals with AD [7]. Excessive mitochondrial fragmentation was described in neurons from AβPP mice [8]. Mitochondrial axonal trafficking deficits and abnormal mitochondrial distribution were detected in transgenic (Tg) amyloid precursor protein (APP) mice [9].

Aβ aggregation may also determine mitochondrial function. Enhanced Aβ burden has been observed in mitochondria from autopsy specimens of late-onset AD [10]. Aβ_1–40_ and Aβ_1–42_ mitochondrial internalization is mediated by the receptor components of the translocase of the outer membrane (TOM), which is independent of the ΔΨm [11]. Several familial and Aβ-based animal models of AD display systemic mitochondrial dysfunction, including decreased COX activity, impaired mitochondrial respiration, augmented glycolysis, and marked oxidative insult. Exposure to Aβ or overexpression of APP resulted in excessive mitochondrial fragmentation and abnormal mitochondrial distribution [12]. Partial reduction in Drp1 protected against Aβ/APP-induced mitochondrial and synaptic impairment. In addition, Aβ can induce S-nitrosylation of Drp1, which increases its translocation into mitochondria [13]. Ca^2+^ signaling and oxidative stress are important contributors to Aβ-induced mitochondrial fragmentation. Aβ promotes mitochondrial Ca^2+^ influx and Drp1-dependent mitochondrial fission via sustained Akt activation [14]. Misfolded tau can disrupt mitochondrial function. Accumulation of tau decreased complex I activity and ATP synthesis and altered mitochondrial dynamics [15]. Complex I immunoreactivity, α-KGDHC and transketolase enzyme activity, and mtDNA copy number were reduced in P301S tau mice [16]. AGEs were attenuated in those mice, which showed an important oxidative and nitrosative damage. Tau can also interact with Aβ to induce a synergistic detrimental effect on mitochondria by exacerbating respiratory capacity abnormalities, downregulating complex I and IV activities, and disturbing energy metabolism. Pathological p-tau exhibits lower affinity for the microtubule network, resulting in increased fission events. Either Tg P301L or P301S tau mice display a perturbed mitochondrial fusion–fission cycle. Tau ablation blocked Aβ-mediated activation of glycogen synthase kinase 3β (GSK3β) resulting in an improvement of the anterograde axonal transport of mitochondria [17].

Sustained mitochondrial dysfunction is a primary cause of an excessive generation of free radicals in AD brains and may lead to Aβ and tau pathology. Aggregation of Aβ or tau within mitochondria not only compromises the function of vital mitochondrial proteins but also increases oxidative damage. The interaction between Aβ_1–40_ and Aβ_1–42_ and the mitochondrion interferes with its function and leads to mitochondrial oxidative damage, reduced COX activity, and increased content of H_2_O_2_ prior Aβ plaque formation. Aβ attenuates mitochondrial respiration and ∆Ψ generation induced by different substrates of complexes I and IV. The Aβ_1–42_ peptide enhances the levels of ROS by inhibiting complex I activity and membrane LPO associated with complex IV deficiencies. Furthermore, a significant increase in the GSSG/GSH ratio was observed in postmortem AD specimens, indicating a defective antioxidant defense system [18]. Free radical-mediated oxidative degradation of PUFAs is a key feature of AD. The LPO product malondialdehyde (MDA) is significantly increased in the cerebral cortex and hippocampus of AD patients [19]. Elevated levels of 4-HNE have been reported in different AD brain regions compared with age-matched controls [20]. Increased 4-HNE immunoreactivity and reduced levels of antioxidant proteins and enzymes were found in the superior temporal gyrus from APOE ε4 cases [21]. Moreover, high levels of 4-HNE and isoprostanes were reported in the cerebral spinal fluid (CSF) samples of AD patients [22]. Redox proteomic analyses revealed a significant lipoxidation and nitration of several key mitochondrial enzymes, including the ATP synthase, in the hippocampus of AD subjects [23,24]. ROS-induced functional variations in the F1Fo-ATP synthase may represent a potential mechanism of OXPHOS deficiency in AD. Protein nitration is also a feature of AD. The number of 3-NT-positive neurons was robustly increased in postmortem brain samples from AD cases [25]. Protein carbonyls are increased in subjects with AD although the expression pattern varies across different brain regions. Carbonylation of proteins and AGE plasma levels were specifically elevated in male AD patients [26]. The content of DNA strand breaks was higher in AD brains compared to controls. HPLC analysis showed a significant increase in 8-OHdG levels in DNA isolated from the brain of idiopathic AD cases, while elevated 8-OHG immunoreactivity was found in the cerebral cortex of preclinical early-onset patients with AD [27].

## 3. Ca^2+^ Dysregulation and Downstream Effects in Alzheimer’s Disease

**Figure 1:**
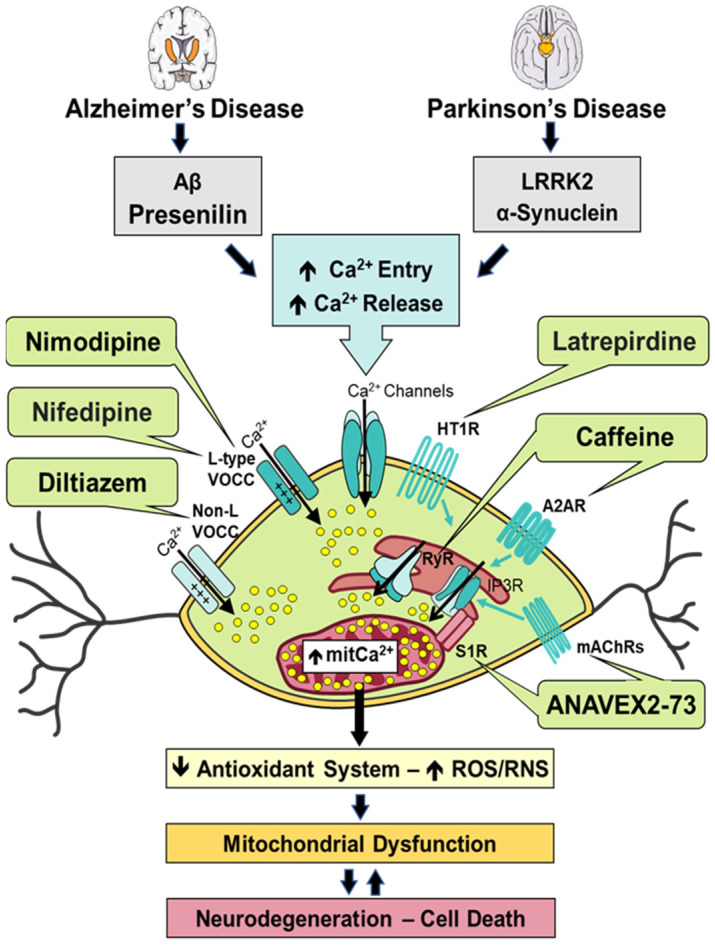
Heterocycles target Ca^2+^ dyshomeostasis leading to mitochondrial Ca^2+^ overload, oxidative damage, and neuronal degeneration in AD and PD.

Neuronal Ca^2+^ signaling is mediated by activation of plasma membrane channels, including voltage-gated Ca^2+^ channels (VOCCs), receptor-operated Ca^2+^ channels (ROCCs), and Ca^2+^-permeable channels, such as transient receptor channels (TRP) or store-operated channels (SOCs). The opening of these channels induces Ca^2+^ influx with the electrochemical gradient, which is also significantly influenced by a large array of voltage-gated K^+^ channels that normally set the mitochondrial potential in resting conditions (Figure 1). Ca^2+^ signaling is also linked to the activation of Ca^2+^ release channels in the ER, including IP_3_ receptors. Release of Ca^2+^ from IP_3_-sensitive stores occurs upon the activation of G protein-coupled receptors (either glutamate metabotropic or acetylcholine muscarinic receptors) that activate phospholipase C. Moreover, activation of ryanodine receptor channels (RyRs) stimulates Ca^2+^ release. These channels can be activated by several messengers (including Ca^2+^ itself) to amplify their signaling through a Ca^2+^-induced Ca^2+^ release (CICR) mechanism. CICR mechanisms increase cytosolic free intracellular Ca^2+^ concentration ([Ca^2+^]_cyt_). This process is limited by endogenous Ca^2+^ buffers, particularly Ca^2+^-binding proteins (e.g., calbindin) and Ca^2+^ extrusion systems, such as plasma membrane Ca^2+^ ATPases (PMCAs), sarcoplasmic and ER Ca^2+^ ATPases (SERCAs), and plasma membrane and Na^+^/Ca^2+^ exchangers (NCXs) that remove Ca^2+^ from cytosol to the extracellular space or intracellular organelles (mainly ER). Mitochondria also influence Ca^2+^ levels and signaling. Cytosolic Ca^2+^ enters the mitochondrial matrix through the mitochondrial Ca^2+^ uniporter (MCU) complex, a process driven by the ΔΨm. Importantly, subtle modifications in the ΔΨm may considerably modify mitochondrial Ca^2+^ uptake ability [28]. Ca^2+^ uptake by mitochondria activates the Krebs cycle and energy production. However, mitochondrial Ca^2+^ overload causes oxidative stress, mPTP opening, and apoptosis. In contrast to other organelles, mitochondria cannot store Ca^2+^ for prolonged periods, releasing it back to the cytosol in a slow fashion through the Na^+^–Ca^2+^ exchanger (NCLX).

Ca^2+^ hotspots or microdomains are small clusters of high concentration of Ca^2+^ located in so-called mitochondria-associated membranes that efficiently activate the low-affinity Ca^2+^ channel MCU. Only mitochondria in close proximity to Ca^2+^ channels, such as IP_3_ receptor channels at the ER and other Ca^2+^ channels at the plasma, can efficiently import Ca^2+^. We used mitochondria-targeted aequorin, which limits mitochondrial Ca^2+^ overload involved in mPTP, to monitor the coupling of Ca^2+^ release and mitochondrial Ca^2+^ uptake in the soma and neurites of neurons [29,30]. However, Ca^2+^ overload may occur following alterations in Ca^2+^ influx, release, buffers, or extrusion systems, leading to neuronal death. Changes in intracellular Ca^2+^ homeostasis, including variations in resting levels of intracellular Ca^2+^ and storage, have been reported in AD. Mutations in the gene encoding presenilins (PS) 1 and 2, which process APP either directly or indirectly via Aβ production, can alter Ca^2+^ store content and release. The mechanism via which Aβ species induce neuronal damage is not totally understood. It has been proposed that Aβ proteins can be integrated in lipid bilayers to form Ca^2+^-permeable pores termed amyloid channels. We showed that Aβ_1–42_ oligomers increase Ca^2+^ influx in hippocampal neurons, followed by mitochondrial Ca^2+^ overload as monitored by bioluminescence imaging in individual neurons expressing mitochondria-targeted aequorin [31]. Mitochondrial Ca^2+^ overload induced by Aβ_1–42_ oligomers promotes mPTP opening and subsequent CYT C release and apoptosis. A neuroprotective effect was observed after blocking mitochondrial Ca^2+^ uptake without affecting the increase in cytosolic Ca^2+^ levels. In contrast, small mitochondrial depolarization dissipated the driving force for mitochondrial Ca^2+^ uptake, resulting in diminished mitochondrial Ca^2+^ overload, release of CYT C, and apoptosis [31].

Administration of compounds targeting MCU or ΔΨm may confer protection against Aβ oligomer-induced pathology. Therefore, mitochondrial Ca^2+^ has become an attractive target for the development of novel AD agents. Aging plays an important role in Ca^2+^ homeostasis and signaling. We developed a model of neuronal aging based on long-term culture of hippocampal neurons that, under specific conditions, acquire an aging phenotype. We showed that VOCCs-mediated Ca^2+^ responses following plasma membrane depolarization or toxin exposure, including the glutamate receptor agonist NMDA and Aβ oligomeric forms, were significantly elevated in aged neurons compared to young neurons [32,33]. These functional changes were related to modifications in the expression of NMDA receptor (NMDAR) subunits. Our results suggest a critical role of mitochondrial Ca^2+^ in neuronal death. Furthermore, our findings demonstrated that aged neurons undergo significant changes in the expression and/or activity of specific molecular players involved in Ca^2+^ transport, which renders them more vulnerable to damage. Long-term culture of hippocampal neurons underwent Ca^2+^ remodeling [34]. Aged neurons displayed a high resting [Ca^2+^]_cyt_, as well as Ca^2+^ store and release from the ER, simultaneously with an increased Ca^2+^ trafficking from the ER into mitochondria. Aged neurons exhibited a significant decrease in the store-operated Ca^2+^ entry (SOCE), a pathway related to dendritic spine stability and memory storage. Ca^2+^ homeostasis disruptions found in aging neurons may promote energy production but with the potential risk of increased susceptibility to mitochondrial Ca^2+^ overload, reduced spine stability, and neuronal loss. There is a correlation between these functional changes and altered expression of the IP_3_, NMDA, and TLR4 receptors, the mitochondrial MCU, and the plasma membrane molecular players involved in SOCE [35]. Treatment of aged neurons with Aβ oligomers exacerbated many changes involved in Ca^2+^ remodeling.

The mechanism via which Aβ peptide species, particularly oligomers, cause neuronal damage remains unknown. It has been suggested that Aβ species form Ca^2+^ permeable pores or channels responsible for Ca^2+^ entry. However, a more widely accepted hypothesis proposes that Aβ oligomers bind to plasma membrane Ca^2+^ channels, particularly NMDAR channels. We showed that Aβ oligomer-mediated Ca^2+^ responses were highly dependent on synaptic networking [36]. Generation of neural spontaneous, synchronous [Ca^2+^]_cyt_ oscillations were abolished by different antagonists of synaptic transmission (NMDAR) and antagonists of action potential propagation (tetrodotoxin). Our results suggest the involvement of both NMDAR and amyloid channels in the primary response to Aβ oligomers, further enhanced by networking activity. In support of this notion, we also demonstrated that non-neuronal cells expressing NMDAR exhibit Ca^2+^ responses to Aβ oligomers, in contrast to cells lacking NMDA. Expression of NMDAR subunits NR1/NR2A and NR1/NR2B in these cells restored Ca^2+^ responses to NMDA but not to Aβ oligomers, indicating that Aβ oligomers may promote Ca^2+^ entry via amyloid channels and NMDAR. Aβ oligomers may also activate distant neurons intertwisted by synaptic connections, spreading excitation by recruiting additional NMDAR and specific VOCCs, and leading to excessive Ca^2+^ entry, excitotoxicity, and neuron degeneration in AD [36]. Since dysregulated intracellular Ca^2+^ homeostasis plays a pivotal role in the susceptibility to neuron damage during aging, Aβ burden, PS mutations, and excitotoxicity, we are currently working on the development of novel drugs targeting these pathways, specifically in the synthesis of marine heterocyclic compounds [37].

## 4. Impaired Mitochondrial Function and Associated Oxidative Damage in Parkinson’s Disease

PD is a chronic neurodegenerative disease characterized by the loss of DA neurons in the substantia nigra (SN) and their axonal projections to the striatum. A histopathological hallmark of the disease is the presence of neuronal cytoplasmic inclusions termed Lewy bodies (LB), which are predominantly composed of α-syn and, to a lesser extent, ubiquitin. *SNCA* and *LRRK2* gene point mutations or multiplications cause familial dominant PD. It has been suggested that perturbed glucose metabolism is an early event in idiopathic PD. Using [^18^F]-fluro-2-deoxyglucose PET imaging, glucose hypometabolism was detected in the cerebral cortex of individuals with early-stage PD and the hippocampus and in the temporo-parietal and occipital regions of PD dementia (PDD) patients [38]. Mitochondrial defects in PD also involve morphologically abnormal mitochondria. In SN DA neurons of patients with PD, ~80% of total mitochondria exhibited an irregular shape and swollen morphology with deranged cristae patterns. Using electron microscopy, subsets of mitochondria appeared swollen and rounded or enlarged in cybrid cells prepared from platelet-derived mtDNA of sporadic PD cases [39]. In contrast to AD, the levels of thiamine remain unchanged in the plasma of PD cases. Nevertheless, free thiamine content was significantly reduced in CSF specimens of PD subjects [40]. α-KGDH enzymatic activity declined by 50% in PD patients [41]. However, increased α-KGDH immunoreactivity was detected in the SN of patients with idiopathic PD [42]. Downregulated activity of α-KGDH, PDHC, and succinate dehydrogenase (SDH) parallel to mitigated respiratory function was observed in PC12 cells overexpressing monoamine oxidase B (MAO B) [43].

Systemic deficiencies in complex I assembly and decreased activity might result in impaired oxidative capacity, ensuing ROS/RNS overproduction, and progressive mitochondrial deficiencies, a major culprit in the degenerative process of DA neurons. Reduced complex I activity was reported in SN tissue of postmortem human samples [44,45]. Impaired catalytic activity of complex I has been found in the frontal cortex and in peripheral tissues such as, skeletal muscle, platelets, and lymphocytes of late-onset PD subjects. Mounting evidence supports the notion that defects in mitochondrial dynamics are likely a common mechanism leading to mitochondrial dysfunction and neurodegenerative process in PD. OPA1 immunoreactivity was significantly attenuated in the SN tissue from patients with sporadic PD [46]. OPA1 is further linked to non-syndromic, idiopathic PD associated with aberrant changes in cristae structure and disrupted mitochondrial networks [47]. Altered trafficking along axons may represent a slow but steady feature disrupting mitochondrial homeostasis in PD, since mitochondria undergo bidirectional transport along microtubule and actin filaments. SN DA neurons displayed low levels of kinesin heavy chain (KHC) and kinase light chain (KLC1) in subjects with early-onset PD, while the DYNLT3 level decreased in patients with late-onset PD [48]. Parkinsonized rats exhibited decreased content in mitochondrial fusion proteins, increased levels of DRP1, and attenuated anterograde and retrograde axonal transport [49,50]. α-Syn localizes not only in the cytosol but also at or in mitochondria of DA neurons in different systems, including patients with PD. Mitochondria-targeted α-syn may cause structural damage to the organelle. Expression of α-syn compromised mitochondrial structural integrity, including massive swelling, abnormal morphology, and distorted cristae. Accumulation of α-syn led to a threefold decrease in complex I activity in the SN of postmortem PD brains [51]. Selective reduction in complex I immunoreactivity was observed in midbrain homogenates of AAV-A53T α-syn-transduced rats [50]. Inhibition of complex I activity and altered levels of complex I-related proteins were observed following α-syn mitochondrial import, which can further increase endogenous content of α-syn, thereby initiating a feedforward cycle.

α-Syn plays an important role in controlling mitochondrial integrity by regulating dynamics, transport, and clearance. A53T and A30P mutations in α-syn elicited mitochondrial fragmentation via a DRP1-independent pathway and increased OPA1 cleavage [52]. In a different study, α-syn-induced mitochondrial fragmentation occurred via a Drp1-independent or -dependent mechanism [53]. The authors also showed that overexpression of MFN1 or MFN2 did not prevent fragmentation, supporting the idea that α-syn plays a selective role in fission events. α-Syn-induced mitochondrial fragmentation precedes OXPHOS disturbances, including mitochondrial respiration and ΔΨm [53]. Preformed α-syn fibrils promoted mitochondrial fission in DA neurons [54]. A53T mutant α-syn increased the amount of short round-shaped (fragmented) mitochondria, supporting a role for α-syn in regulating mitochondrial morphology, probably linked to an association between the N-terminal region and the mitochondrial membrane [55]. Synthetic α-syn fibrils can cause axonal transport deficiencies, with variations in kinesin and dynein markers [54]. Overexpression of α-syn (especially A53T mutation) led to alterations in mitochondrial flux, suggesting an imbalance in mitochondrial distribution along axons. α-Syn can gradually spread to more rostral brain regions and accumulate in dystrophic axons, where it is distributed and propagated in both ipsilateral and contralateral sides via axonal transport [56]. In addition, ΔΨm is required for α-syn transport and is dependent on the ATP pool. α-Syn can also promote Ca^2+^ trafficking from the ER to the mitochondria via mitochondria-associated ER membranes. α-Syn increases OH^•^ concentration and protein thiol oxidation in DA neurons [51,57]. Moreover, knockdown of endogenous α-syn expression resulted in a robust reduction of protein thiol oxidation in SN DA neurons. Fibrillated α-syn augmented ROS/RNS production by increasing the MitoSox (O_2_^•–^) signal and the levels of 3-NT in DA neurons [54]. AAV-driven overexpression of human mutated A53T α-syn into the rat SN elicited systemic oxidative insult, including LPO and protein nitration in the ipsilateral hemisphere [50].

## 5. Altered Ca^2+^ Homeostasis and Concomitant Neurotoxicity in Parkinson’s Disease

SN DA neurons are under extreme bioenergetic demand because of three particular features: (i) the maintenance of a massive axonal tree that requires anterograde/retrograde transport of cytosolic metabolites and organelles, (ii) the maintenance and propagation of action potentials (APs) and the restoring of ionic gradients, and (iii) the large number of synaptic vesicles cycling. DA neurons display large [Ca^2+^]_cyt_ oscillations that can promote mitochondrial OXPHOS activity, which may render them vulnerable to stressor-mediated damage, including genetic mutations, mitochondrial toxins, and defective aging (Figure 1). Surmeier et al. proposed that DA neurons exhibit Cav1.3 L-type Ca^2+^channel-dependent pacemaking activity. Reliance of pacemaking activity on these VOCCs increased in an age-dependent manner, suggesting that mature SN DA neurons might use alternative mechanisms for pacemaking activity common to other neuron types unaffected in PD [58]. A plausible mechanism involves that juvenile mechanisms remain latent in aged neurons, thereby protecting DA neurons from degeneration. Treatment with isradipine, a Cav1.3-selective L-type Ca^2+^ channel antagonist, can reverse the pacemaking phenotype and protect DA neurons against toxin-induced parkinsonism.

Spike-activated Ca^2+^ influx mediated by Cav1.3 channels may trigger Ca^2+^ release from the ER, which is imported into the mitochondria to stimulate OXPHOS activity via two Ca^2+^-dependent mechanisms: (i) a MCU-dependent Ca^2+^ uptake mechanism that contributes to boost the Krebs cycle and mitochondrial respiration; (ii) a mechanism dependent on the malate–aspartate shuttle system that translocates electrons generated during the glycolysis across the semipermeable mitochondrial inner membrane. However, if these mechanisms are disrupted, the ability of DA neurons to sustain a spike activity is impaired. This feedforward mechanism sustains the bioenergetic demands of DA neurons, which is associated with oxidative stress and concomitant degenerative process. Epidemiological studies provided evidence of a significant correlation between low PD risk and the use of Cav1 channel inhibitors such as dihydropyridines. A phase III clinical trial showed that chronic administration of isradipine did not slow clinical progression of early-onset PD [59]. A potential explanation for isradipine failure in clinical trials may include the extensive DA neuron degeneration in patients at the time of diagnosis or recruitment. Another reason arises from the low isradipine concentration achieved in the central nervous system (CNS) due to large drug clearance. Despite this failure, there is still a substantial interest in developing novel drugs selectively targeting Ca^2+^ signaling in DA neurons.

## 6. Modulation of Calcium Signaling and Homeostasis by Heterocyclic Compounds in Alzheimer’s Disease and Parkinson’s Disease

Extensive evidence has demonstrated that the dysregulation of Ca^2+^ homeostasis and signaling is a mechanism that plays an essential role in the etiopathogenesis of AD and PD. Aβ peptides are involved in the excessive accumulation of intracellular Ca^2+^ through the formation of Ca^2+^-permeable pores in the plasma membrane or by increasing Ca^2+^ influx via activation of L-type VOCCs and NMDA or AMPA receptors. Genetic mutations in PS or activation of inositol 1,4,5-trisphosphate (IP_3_) and RyRs promote Ca^2+^ leak in the ER, enhancing the concentration of both cytosolic and mitochondrial Ca^2+^. Knockdown or inhibition of IP_3_ kinase B led to α-syn aggregation and phosphorylation in cortical neurons treated with α-syn fibrils, which increases (i) Ca^2+^ release from the ER, (ii) mitochondrial Ca^2+^ content, and (iii) mitochondrial respiration [60]. Moreover, mutations in the gene encoding for LRRK2 elicited transcriptional upregulation of the MCU and the mitochondrial Ca^2+^ uptake 1 protein (MICU1) in postmortem human PD brains, fibroblasts, and mice [61]. These findings implicate mitochondrial Ca^2+^ dyshomeostasis in the pathogenesis of AD and PD. Herein, we review some heterocyclic compounds with the capability of modulating Ca^2+^ signaling that can be considered as promising pharmacological agents for the treatment of neurodegenerative diseases (Figure 1).

### 6.1. ANAVEX2-73

ANAVEX2-73 (Blarcamesine) is a nonselective muscarinic acetylcholine receptor (mAChR) and sigma-1 receptor (S1R) ligand that exhibits an important affinity for its pharmacological targets at micromolar concentration. σ1 receptors are ubiquitously expressed in the CNS and are located at mitochondria-associated ER membranes, where they interact with IP_3_ receptors to regulate Ca^2+^ exchange between the ER and mitochondria, thereby resulting in reduced mitochondrial stress, regulation of ion channels, activation of the nuclear erythroid 2-related factor 2 (NRF2)/antioxidant response element (ARE) pathway, and limited apoptosis. IP_3_ receptor-mediated Ca^2+^ release correlates with variations in the availability of mitochondrial Ca^2+^ and ATP synthesis. A formal concept analysis (FCA) combined with the knowledge extraction and management (KEM) environment was able to identify ANAVEX2-73 as a potential genomic biomarker of disease and therapeutic response in a phase IIa clinical trial [62]. In a double-blind, placebo-controlled phase IIb/3 clinical study, ANAVEX2-73 improved cognition (ADAS-Cog) and function (ADCS-ADL) in patients with early AD (ANAVEX2-73-AD-004). Intraperitoneal injection of ANAVEX2-73 reversed scopolamine- and dizocilpine-induced learning impairments after intracerebroventricular injection of the neurotoxic Aβ_25–35_ peptide in mice [63]. Treatment with ANAVEX2-73 inhibited the phosphatidyl-inositol 3-kinase (PI3K)/Akt pathway, thereby resulting in the activation of GSK-3β, improved behavioral deficits, and reduced Aβ seeding and tau-induced pathology in Aβ_25–35_-injected mice [64].

ANAVEX2-73 also preserved mitochondrial integrity and function by increasing the activity of complex IV and oxygen consumption at all states [65]. Exposure to ANAVEX2-73 also mitigated the peroxidation of lipids, Bax/Bcl-2 ratio, and CYT C release. Administration of ANAVEX2-73 restored mitochondrial respiration and preserved complex IV activity levels from Aβ toxicity in a Ca^2+^-dependent fashion [66]. ANAVEX2-73 displayed a synergic effect with donepezil (but not with memantine) and restored spontaneous alternation and passive avoidance response in a non-Tg mouse model of AD [67]. Administration of ANAVEX2-73 to Aβ_42_-expressing *C. elegans* upregulated proteostasis capacity, resulting in a dissociation and clearance of Aβ_42_ aggregates [68]. In nematodes, ANAVEX2-73 stimulated autophagic activity. In summary, ANAVEX2-73 interacts with IP_3_ receptors to modulate Ca^2+^ release and to prevent mitochondrial failure in multiple ways, including through the activation of the NRF2/ARE pathway that directly controls the expression of several antioxidant and anti-inflammatory genes. In addition, ANAVEX2-73 promotes autophagosome biogenesis and autophagic flux to degrade aggregated proteins and damaged organelles. Clinical benefits (MDS-UPDRS and CGI-I) were reported in a 48-week phase II extension study in patients with PDD (NCT04575259). An ongoing randomized, double-blind phase III trial of this compound is evaluating its bioavailability and the safety, tolerability, and effectiveness of the treatment in patients with AD (NCT03790709), PD with dementia (NCT03774459), and Rett syndrome (NCT03758924).

**Scheme 1:**
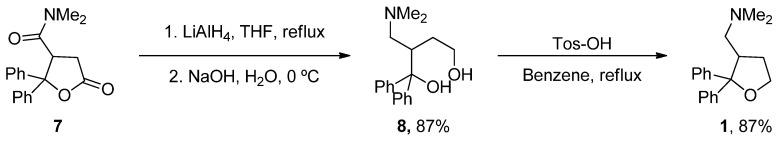
Unique total production of ANAVEX2-73 **1**.

The unique synthesis of ANAVEX2-73 (1-(2,2-diphenyltetrahydro-3-furanyl)-*N*,*N*-dimethylmethanamine hydrochloride) **1** was reported by Foscolos et al. [69]. The key step during the production of ANAVEX2-73 is the reduction opening of lactone **7** to provide 1,4-diol **8**, which is subsequently converted by acid-catalyzed cyclodehydration to ANAVEX2-73 **1** (Scheme 1).

### 6.2. Caffeine

Caffeine (mateine), a purine alkaloid present in several plants, is the most consumed psychoactive substance in the world. Coffee is a rich source of bioactive components that contribute to its biological activity, including potassium (K^+^), magnesium (Mg^2+^), niacin, and potent antioxidants. Caffeine alters Ca^2+^ signaling and promotes its release in a process independent of extrusion mechanisms or variations in the steady-state concentration of specific Ca^2+^-binding proteins. This effect was attributed to the activation of RyRs. Chronic caffeine intake promoted CSF production combined with an elevated cerebral blood flow velocity and Na^+^/K^+^-ATPase levels (which play an important role in memory and learning) [70]. Administration of caffeine upregulated protein kinase A (PKA) activity, induced cyclic adenosine monophosphate (cAMP)-response element binding protein (CREB) phosphorylation, and reduced the levels of phosphorylated c-Jun N-terminal kinase (JNK) and extracellular signal-regulated kinase (ERK), suggesting that caffeine induced proapoptotic signaling [71]. Caffeine intake resulted in memory capacity improvement and increased brain neurotrophic derived factor (BDNF) and tyrosine receptor kinase B (TrkB) levels [72]. Research studies have established a strong relationship between frequent caffeine consumption and reduced risk of developing AD and PD, with no detectable adverse effects in the CNS. A mendelian randomization study found that genetically predicted higher caffeine plasma content correlates with diminished risk of AD [73]. Following confounding adjustment, long-term coffee consumption (≥2 cups/day) was associated with a significant improvement in cognitive decline [74]. The CAIDE longitudinal epidemiological study established an association between mid-life moderate coffee (but no tea) consumption and late-life reduced risk of dementia and AD [75]. Long-term treatment with caffeine improved behavioral outcome and decreased the levels of Aβ, PS1, and β-secretase (BACE) in Tg APPsw mice [76]. Pathological PS induce Ca^2+^ accumulation and release, in part via its biochemical interaction with the IP_3_ receptor, thereby resulting in an anomalous regulation of Ca^2+^ signaling pathways and the stimulation of APP processing and Aβ synthesis, even prior to the formation of plaques and NFTs [77]. Brain and plasma Aβ burden and enhanced memory performance were observed in APPsw and APP/PS1 mice following either acute or chronic caffeine administration [78]. Oral supplementation with caffeine restored motor performance, attenuated anxiety and memory deficits, prevented neuronal death in the CA1 pyramidal layer of the hippocampus, and promoted neurogenesis in both 5xFAD and Tg4-42 mouse models of AD [79]. Caffeine attenuated spatial memory abnormalities, tau phosphorylation, oxidative stress, and inflammation in THY-Tau22 mice [80].

The Honolulu Heart Program provided the first evidence of a potential beneficial effect of caffeine intake in PD patients. Coffee drinkers (≥28 oz/day) had fivefold lower incidence of developing PD following an adjustment for both age and pack-years of cigarette smoking [81]. This study was further supported by Ascherio et al. that carried out a prospective study of caffeine consumption and risk of PD in men and women [82]. According to an open-label, dose-escalation study, caffeine had potential motor and nonmotor benefits in subjects with PD, with the maximum tolerated dose of 100–200 mg/day bis in die (BID) without affecting sleep quality [83]. In a randomized controlled trial, administration of 200 mg caffeine BID for 6 weeks did not improve daytime somnolence but had the potential to reverse motor symptoms in PD patients [84]. Caffeine consumption mitigated the risk of PD, but its neuroprotective properties may vary depending on its interaction with other factors, such as obesity and low serum cholesterol levels [85]. A meta-analysis involving a large number of participants found a nonlinear relationship between coffee consumption and the incidence of PD that achieved the maximum protective effect at three cups per day [86]. However, there was a linear dose relationship of reduced risk of PD with both tea and caffeine consumption, especially in men compared to women and in the European and Asian population relative to USA residents. There was a negative correlation between caffeine intake and risk of developing PD in men but a U-shaped relationship in women. Interestingly, caffeine diminished the risk of PD in menopausal women that did not take hormone replacement, but there was a higher risk (fourfold) among hormone users with high caffeine [87]. The risk of PD was even lower when coffee intake was combined with cigarette smoking and nonsteroidal anti-inflammatory drug use, resulting in a cumulative effect [88].

Administration of caffeine provided a dose-dependent therapeutic effect against rotenone-mediated neurotoxicity. Injection of caffeine through a peritoneal route improved the behavioral phenotype, normalized brain enzymatic activities of both acetylcholinesterase (AChE) and Na^+^/K^+^-ATPase, and mitigated oxidative damage and neuroinflammation in rotenone-treated rats [89]. Caffeine modulated the levels of CYT P450, glutathione-S-transferases, and vesicular monoamine transporter 2 (VMAT-2) in mice receiving 1-methyl-4-phenyl-1,2,3,6-tetrahydropyridine (MPTP) [90]. Pretreatment with caffeine increased neuronal glutamatergic and GABAergic metabolic activity and neurotransmission in MPTP-injected mice [91]. Exposure to caffeine also improved motor dysfunction and increased catecholamine levels in a 6-hydroxydopamine (6-OHDA) rat model of PD [92]. Striatal injection of 6-OHDA elicited apomorphine-induced rotation and impaired locomotor activity in parallel with a loss of DA immunoreactivity and an inflammatory response in rats, but administration of caffeine ameliorated the behavioral and pathological PD-like phenotype. Moreover, caffeine attenuated autophagy in mice injected with α-syn fibrils [93]. Regarding its potential mechanism of action, caffeine is an antagonist of the adenosine-2A receptor (A2AR), predominantly located in the GABAergic striatopallidal neurons projecting from the caudate nucleus and the putamen to the external segment of the globus pallidus. A2ARs colocalize with DA2 receptors to form heteromeric complexes that mediate the allosteric antagonism between adenosinergic and DAergic transmission. In summary, while it has complex biological and pharmacological profiles, evidence indicates that caffeine readily crosses the blood–brain barrier, exerts its action by antagonizing A2ARs, and confers neuroprotection by stimulating mitochondrial function and by attenuating excitatory neurotransmitter release, oxidative insult, and neuroinflammation. In addition, caffeine has a well-established long-term safety profile. Together with its low cost and high availability, caffeine is a promising therapeutic agent for the treatment of neurodegenerative diseases.

**Scheme 2:**
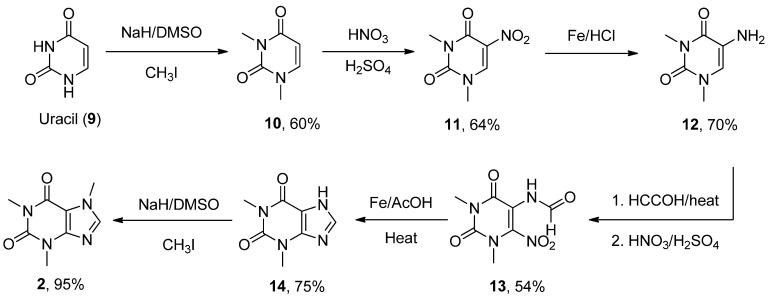
Synthesis of caffeine **2** from uracil **9**.

The most recent synthesis of caffeine (1,3,7-trimethyl-3,7-dihydro-1*H*-purine-2,6-dione) **2** was reported by Narayan et al. and required seven reaction steps [94]. N-Methylation of uracil **9** in the presence of a strong base such as sodium hydride in dimethylsulfoxide (DMSO) produced 1,3-dimethyluracil **10**, which was nitrated and subsequently reduced to 5-amino-1,3-dimethyluracil **12**, using iron and hydrochloric acid. Nitro derivative **13** was obtained following two conventional steps and led to theophylline **14** formation after a catalytic reduction step and an intramolecular heterocyclization reaction with iron and acetic acid. The final methylation at position 7 led to the generation of caffeine **2**. The overall reaction yield (amount of product generated by a chemical reaction) was 10% (Scheme 2).

**Scheme 3:**
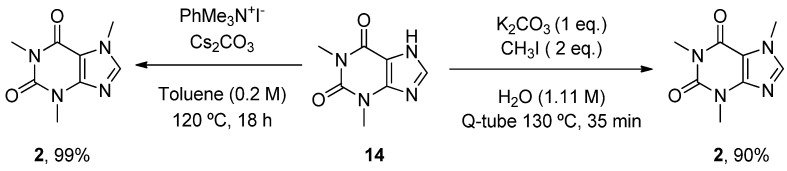
Novel approaches for the N-methylation of theophylline **14**.

New and improved methodologies for the N-methylation of theophylline **14** have been reported. A novel technique used the Q-tube system in which reactions were carried out under high pressure, overcoming the solvent boiling point. Consequently, there was a significant increase in the pressure and temperature, leading to a decreased reaction time [95]. Water was used as a green solvent, and the target compounds were achieved generally in good yields. In a different study, Schnürch’s group optimized the quaternary ammonium salts as the methylating agents. Specifically, the authors developed a new protocol for monoselective methylation and ethylation of amides, indoles, and related structures [96] (Scheme 3).

### 6.3. Diltiazem

Diltiazem (Cardizem) is a non-dihydropyridine Ca^2+^ channel blocker with antihypertensive, antiarrhythmic, and vasodilation properties. The drug selectively targets VOCCs, which are the primary mediators of Ca^2+^ influx into neurons in response to membrane depolarization. P/Q- and N-type VOCCs regulate neurotransmitter release upon arrival of the action potential to the axon terminal in the presynaptic neuron. Glutamate release promotes postsynaptic Ca^2+^ trafficking by activation of NMDAR or through an indirect pathway involving L-type VOCCs. Reduced transient Ca^2+^ at presynaptic or postsynaptic sites can mitigate glutamate-induced excitotoxicity. Therefore, administration of Ca^2+^ channel blockers has become an interesting approach for the treatment of neurodegenerative diseases. To the best of our knowledge, no clinical trials have been conducted studying the effects of diltiazem on patients with AD. Experimental evidence showed that exposure to diltiazem blocked Aβ_25–35_-induced AChE activity through modulation of the L-type VOCCs [97]. Diltiazem protected neurons from Aβ-mediated influx of Ca^2+^ ions and downstream toxicity, as well as facilitated Aβ clearance rate [98]. Treatment with diltiazem improved survival rate and decreased intracellular Ca^2+^ concentration by blocking L-type Ca^2+^ channels in vitro. Augmented APP secretion with no variations in the amount of cell-associated APP occurred in response to diltiazem [99]. Administration of diltiazem improved cell viability and proliferation following exogenous Aβ accumulation, which showed an early Ca^2+^-independent and a late Ca^2+^-dependent phase [60]. Chronic exposure to aluminum or any other of its forms in the drinking water has been associated with higher risk of developing AD. Aluminum accumulation has been detected in the brain of AD cases, which causes neurofibrillary degeneration in neurons. Increased content of aluminum within NFTs was observed in both the inferior temporal cortex and hippocampus of individuals with AD [100]. Aluminum chloride has been reported to cause dementia, but administration of diltiazem reversed learning and memory deficits, AChE upregulation, and oxidative damage in mice [101].

The results of a single clinical trial found no association between diltiazem and the risk of developing PD (<7% of cases) [102]. Cav1.2 and Cav1.3 are L-type VOCCs that regulate DAergic neuron spontaneous firing activity in the SN region of the brain [58]. DA neurons fire either in a low-frequency single-spike pattern or, transiently, in a high-frequency so-called burst mode. Cav1.2 and Cav1.3 L-type VOCCs are susceptible to degeneration during the progression of PD [103]. The extended opening of L-type VOCCs throughout autonomous pacemaking induces prolonged cytoplasmic Ca^2+^ trafficking and its overload in SN DA neurons, leading to mitochondrial oxidative stress [103]. L-type VOCCs-mediated alteration of the steady-state DA levels was responsible for causing downstream oxidative stress and α-syn-induced toxicity [104]. Despite a lack of research investigating the potential antioxidant effect of diltiazem in AD or PD, it has been found that its intraperitoneal injection decreased the levels of nitrite and MDA and upregulated the activity of several antioxidant enzymes [105]. Another study confirmed a significant reduction in MDA content following diltiazem treatment [106]. Oral supplementation with diltiazem reduced the levels of thiobarbituric acid-reactive species (TBARS) and nitrite together with an upregulation of superoxide dismutase (SOD) and reduced glutathione enzymatic activities in an aluminum chloride mouse model of AD [101]. In summary, the L-type VOCC diltiazem is a Ca^2+^ antagonist that can be used to treat dementia and dementia-related diseases due to its capability to improve cognitive deficits, such as learning and memory capacity. In addition, epidemiological data obtained from individuals with PD indicate that long-term treatment with Ca^2+^ channel blockers targeting Ca^2+^ channels of DA neurons may represent a potential therapeutic strategy.

**Scheme 4:**
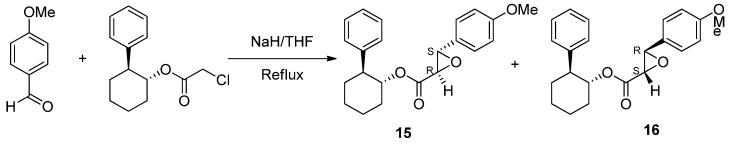
Schwartz’s strategy for the synthesis of diltiazem **3**.

The first racemic production of diltiazem (2-(2-dimethylaminoethyl)-5-(4-methoxyphenyl)-3-oxo-6-thia-2-azabicyclo[5.4.0]undeca-7,9,11-trien-4-yl]ethanoate) **3** was described in 1990 [107]. The first asymmetric synthesis of diltiazem was performed using a diastereoface differentiating nucleophilic addition as the key step to create the two contiguous stereogenic centers [108]. Schwartz used the separation of diastereomeric glycidic esters (**15** and **16**) by direct crystallization, based on their marked difference in solubility [109]. Enantiomerically pure glycidic ester **15**, the required isomer for the synthesis of diltiazem **3**, was the major product obtained (54% yield) (Scheme 4).

**Scheme 5:**
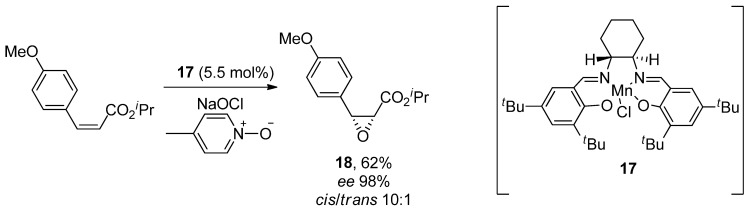
Enantiomerically pure key intermediate **18** for diltiazem **3** production.

In 1994, Jacobsen et al. used a manganese-catalyzed asymmetric epoxidation of cinnamate esters for the preparation of an enantiomerically pure key intermediate **18** in the synthesis of **3** [110] (Scheme 5).

**Scheme 6:**
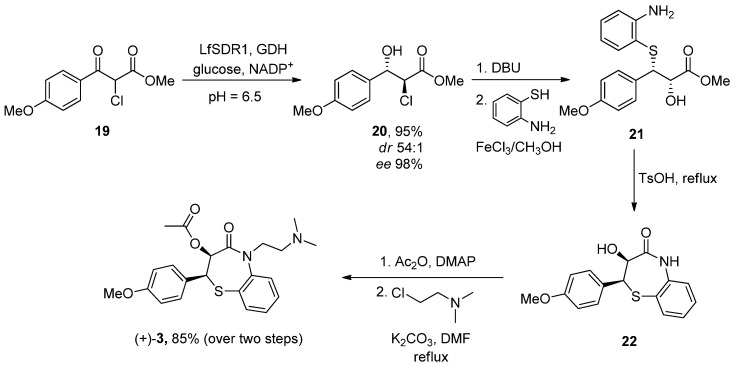
Chemoenzymatic synthesis of diltiazem **3**.

Additional synthetic routes to generate diltiazem **3** from a chiral epoxide intermediate have been reported. The enantiomerically pure epoxide can be obtained through the introduction of a chiral auxiliary in the reaction, through the addition of metal complexes, or by direct acquisition. A useful methodology employed to induce chirality in the product is the utilization of lipase or Baker’s yeast catalyzed reactions. The most recent approach used for the production of diltiazem **3** was reported by Chen et al. [111]. Racemic keto ester **19** was converted into enantiomerically pure hydroxy ester **20** by ketoreductase-catalyzed dynamic reductive kinetic resolution. Two conventional steps (ring closure and ring opening) were further taken to generate an intermediate **21**, which was readily subjected to intramolecular acid-catalyzed amidation. Two additional steps from intermediate benzothiazepinone **22** were required to obtain diltiazem **3**. This chemoenzymatic synthesis involves eight steps to obtain diltiazem **3**, with an ~45% overall reaction yield (Scheme 6).

### 6.4. Latrepirdine

Latrepirdine (Dimebon) is a carboline that blocks H1 histamine receptor (H1R) activity. A broad spectrum of effects on neurologically relevant targets was proposed. The compound is a potent reversible and competitive inhibitor of both AChE and butyrylcholinesterase, which leads to elevated acetylcholine content and concomitant boosting of cognitive performance. In addition to its anti-histaminic properties, latrepirdine can modulate a wide range of other neurotransmitter receptors, such as DA, serotonin, glutamate, and NMDA. Latrepirdine can also block the activity of L-type VOCCs. To date, 21 clinical trials investigating the neuroprotective efficacy of latrepirdine in AD patients have been reported. A pilot clinical trial carried out in patients with mild-to-moderate AD given latrepirdine for 8 weeks showed an important reduction of neuropsychiatric symptoms and a significant cognitive function enhancement, together with a lack of hematological and biochemical disturbances [112]. Data from a phase II randomized, double-blind, placebo-controlled clinical study showed a significant improvement in cognition, function, and behavioral outcome following 60 mg/day latrepirdine for 26 and 52 weeks in individuals with mild-to-moderate AD with no adverse effects recorded [113]. A meta-analysis study revealed that latrepirdine did not ameliorate overall cognition, but the compound enhanced the neuropsychiatric inventory scale, used to assess psychopathology in dementia patients [114]. CONNECTION and CONTACT phase III trials (NCT00838110) failed to show significant improvement in any primary or secondary outcome measures of cognition in patients treated with latrepirdine.

Exposure to latrepirdine preserved neurons against Aβ-mediated toxicity through inhibition of the L-type VOCCs current. Intraperitoneal administration of latrepirdine reversed learning and memory loss following chronic partial deprivation of cerebral cholinergic functions, which causes dementia [115]. Treatment with latrepirdine ameliorated scopolamine-induced memory impairment in young adult and aged Rhesus macaques [116]. Latrepirdine improved cognitive ability in 5xFAD mice, although it was unable to mitigate Aβ-associated pathology [117]. P301S tau mice consistently performed better on both the inverted grid and the accelerating rotarod tests after latrepirdine treatment [118]. Administration of latrepirdine improved cognitive function although it did not modify Aβ_40_ or Aβ_42_ content, suggesting APP processing as a direct target of the drug [119]. FDG-PET studies revealed an increased cerebral glucose utilization in aged mice in response to latrepirdine exposure [120]. SDH activity, ΔΨm, and ATP synthesis were normalized following latrepirdine administration with no differences in the mtDNA copy number, showing a restorative effect on mitochondrial function [121]. Latrepirdine prevented Aβ-mediated abnormalities in mitochondrial shape, mass, and respiratory chain complex activities. The compound can also preserve mitochondrial function by targeting the mPTP opening [122]. Either Ca^2+^ or the Aβ_25–35_ toxic fragment increased mitochondrial LPO, but exposure to latrepirdine reduced oxidative damage to lipids [122,123]. Latrepirdine diminished the content of hyperphosphorylated tau-positive dystrophic neurons, although no changes in the levels of inflammatory markers were found. Latrepirdine has a pro-autophagic activity. Indeed, treatment with latrepirdine promoted MTOR- and ATG5-dependent autophagy, which results in decreased levels of intracellular APP metabolites, including Aβ [124]. Although several clinical trials have been conducted in individuals with Huntington’s disease, there is a lack of clinical research investigating the symptomatic and functional outcome following latrepirdine monotherapy in PD. Nevertheless, evidence showed that latrepirdine elicits beneficial effects in experimental PD. Administration of latrepirdine attenuated methamphetamine-induced toxicity [60]. Increased lifespan and motor performance parallel to reduced γ-syn aggregation and inflammatory response were observed in Tg mice overexpressing γ-syn treated with latrepirdine [118]. In summary, psychiatric symptoms (such as depression) and learning and cognitive measures were restored in AD patients undergoing latrepirdine treatment, in part due to its ability to increase acetylcholine concentration by inhibition of either AChE or histamine receptors. Latrepirdine may elicit neuroprotective activity by promoting mitochondrial function and clearance of a range of intracellular inclusions through the stimulation of autophagy. The molecule may also regulate several targets involved in neurodegenerative diseases, such as neurotransmitter receptor activity, stabilization of Ca^2+^ signaling, and oxidative stress. Toxicological studies have shown that latrepirdine is a safe and well-tolerated drug. A dosage exceeding the therapeutic range by 100 times for 2 months did not cause any physiological changes or pathology [112].

**Scheme 7:**
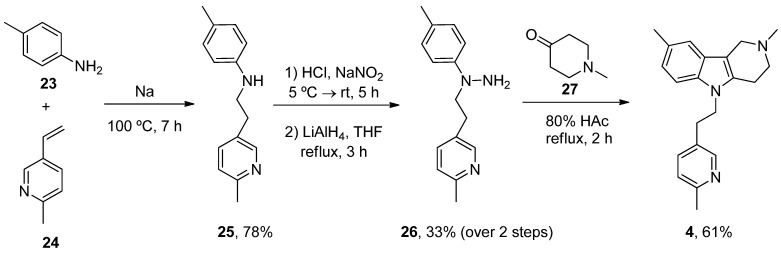
Zheng’s protocol to generate latrepirdine **4**.

Latrepirdine (2,3,4,5-tetrahydro-2,8-dimethyl-5-[2-(6-methyl-3-pyridinyl)ethyl]-1*H*-pyrido[4,3-*b*] indole) **4** was originally synthesized using the Fischer indole reaction. Nevertheless, a more recent synthesis reported by Zheng et al. used *p*-toluidine **23** and 2-methyl-5-vinylpyridine **24** as commercial starting materials and achieved the desired product **4** in four reaction steps, with a ~16% overall reaction yield [125]. Cyclization of 2-methyl-5-(2-(1-(*p*-tolyl)hydrazinyl)ethyl)pyridine **26** and 1-methylpiperidin-4-one **27** was performed at reflux temperature with 80% HAc (Scheme 7).

**Scheme 8:**
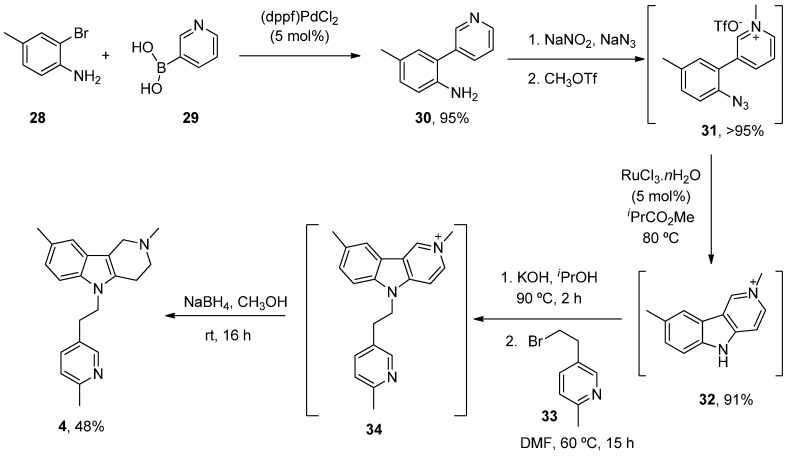
Ruthenium(III) catalysis used in the synthesis of latrepirdine **4**.

Latrepirdine **4** was also produced employing an efficient ruthenium(III) catalysis [126]. The key step involved the stereoselective formation of γ-carboline **32** from *ortho*-substituted aryl azide **31** catalyzed by RuCl_3_·*n*H_2_O. The total synthesis comprised six steps with an ~47% overall yield (Scheme 8).

### 6.5. Nifedipine

Nifedipine (Procardia) is a first-generation dihydropyridine Ca^2+^ channel blocker used to treat hypertension and to control angina pectoris. Nifedipine is a selective antagonist of the L-type VOCCs that plays an essential role in neuronal processes triggered by membrane depolarization, thereby contributing to Ca^2+^-mediated events activated by signaling pathways and diverse stimuli, including neurotransmitter release, rhythmic firing, and gene expression. There is growing evidence that nifedipine may be an effective therapeutic agent for the treatment of neurodegenerative diseases, including AD and PD. Nifedipine counteracts APOE ε4-induced increase of intracellular Ca^2+^ levels and transcriptional activity of CREB, suggesting that L-type VOCCs are involved in neuron responses to APOE ε4. Exposure to nifedipine prevented Aβ-mediated increase of cytosolic Ca^2+^ concentration and synaptic pathology, indicating that Ca^2+^ influx via L-type VOCCs is involved in Aβ toxicity [127]. Up to date, nifedipine has not yet been tested in patients with AD. Administration of nifedipine significantly diminished the content of secreted Aβ_1–42_ and key components of the gamma secretase complex (e.g., PS1) [128]. Treatment with nifedipine promoted the survival rate of CNS neurons cultured from PS1-deficient mice (which showed higher susceptibility to oxidative stress) [129]. Treatment with nifedipine restored post-hypoxic related damage, leading to an improvement of neuron functional deficiencies such as population spike amplitude and depolarized resting ΔΨm in mice lacking APP [130]. Nifedipine blocked the increase in resting [Ca^2+^]_cyt_ concentration in cortical neurons of 3xTg-AD or APPsw mice [131]. An age-dependent increase in L-type VOCC amplitude was described specifically in CA1 pyramidal neurons, consistent with the notion that CA1 neurons are prone to p-tau/NFT pathology due to excessive Ca^2+^ trafficking. Nevertheless, Ca^2+^ current was limited by nifedipine [132]. The stromal interaction molecule 1 (STIM1), a type I transmembrane protein that plays a crucial role in Ca^2+^ influx, negatively modulating L-type VOCCs. STIM1-KO cells displayed an elevated Ca^2+^ entry in response to membrane depolarization, which was nifedipine-sensitive. Hippocampal neurons from 5xFAD mice exposed to nifedipine inhibit abnormal VOCC and store-operated Ca^2+^ (SOC) entry, a process regulated by STIM1. Nifedipine inhibited mutant tau-induced intracellular Ca^2+^ responses to membrane depolarization [133]. Furthermore, administration of nifedipine limited tau-mediated Ca^2+^ influx, generation of ROS, and overall toxicity.

Data derived from a single available clinical study found no evidence of correlation between nifedipine and PD prevalence.[134] However, the results may have been undermined by low statistical power (small sample size). The subthalamic nucleus (STN) has been proposed to play a central role in the disrupted function of the basal ganglia circuitry associated with PD. Distinct activity patterns, such as increased burst firings, have been observed in STN neurons. Treatment with nifedipine caused an irreversible reduction in burst frequency and abolished burst firing [135]. On the basis of nifedipine’s effects on the frequency and current curve, it was established that both short- and long-duration rebound bursting neurons contain nifedipine-sensitive Cav1.2–1.3 channels, which only contribute to rebound activity in STN neurons with long-lasting rebounds. Oscillations produced by high-frequency stimulation in the STN resulted from Ca^2+^ entry through the high-threshold potential nifedipine-sensitive L-type VOCCs [136]. Although nifedipine did not improve DA neuron survival, the drug had a positive impact on neurite length. Stimulation of D1 receptors or the cAMP pathway led to cytosolic accumulation of Ca^2+^ that interacts with nifedipine, resulting in Ca^2+^-mediated CREB phosphorylation and c-fos gene expression [137]. Subcutaneous injection of nifedipine attenuated apomorphine-induced rotation and partially restored striatal DA concentration in 6-OHDA-lesioned rats [138]. Nifedipine prevented nobiletin-induced DA release in MPTP-injected mice [139]. The high-threshold Ca^2+^ spike (HTS) and the slow oscillatory potential (SOP) are diverse sources of Ca^2+^ conductance that play an important role in the generation of action potentials in SN DA neurons. While nifedipine showed a slight inhibitory effect on HTS, the molecule was able to block SOP. Moreover, nifedipine steadily abolished the spontaneous firing pattern [140]. Nifedipine increased cell survival, synaptic vesicle exocytosis, and neurite outgrowth, as well as mitigated DA release and generation of ROS following rotenone exposure [141]. In summary, nifedipine is an antagonist of L-type VOCCs antagonist involved in Aβ and tau pathology, DA neurodegeneration, Ca^2+^ homeostasis and signaling, synaptic function, oxidative insult, and apoptotic cell death. Oral administration of nifedipine had a safety profile at doses of up to 50 mg/kg in rats over a period of 13 weeks. In dogs, no damage was detected up to 100 mg/kg dosage for a period of 4 weeks. Rats tolerated daily intravenous administration of 2.5 mg/kg nifedipine over a period of 3 weeks while dogs tolerated up to 0.1 mg/kg nifedipine for 6 days. An overdose of nifedipine can induce severe hypotension, systemic vasodilation, and reflex tachycardia. 

**Scheme 9:**
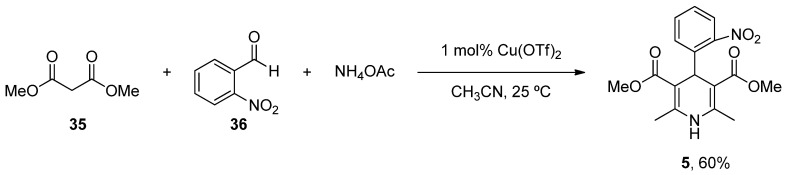
Hantzsch reaction used in the preparation of nifedipine **5**.

The first synthesis of nifedipine **5** (3,5-dimethyl 2,6-dimethyl-4-(2-nitrophenyl)-1,4-dihydropyridine-3,5-dicarboxylate) was reported by Singh et al.[142]using an acid-catalyzed reaction of an enamine with two perhydro-heterocycles. Other synthetic procedures (solid-phase or one-pot solvent-free synthesis) were developed. The most common synthetic procedure to obtain pyridines is the Hantzsch reaction. The preparation of 1,4-dihydropyridines requires two equivalents of β-keto ester, an aldehyde, and a nitrogen donor. Sudalai and coworkers utilized dimethylmalonate **35** as the β-keto ester, 2-nitro-benzaldehyde **36** as the aldehyde, and ammonium acetate as the nitrogen donor [143] (Scheme 9).

**Scheme 10:**
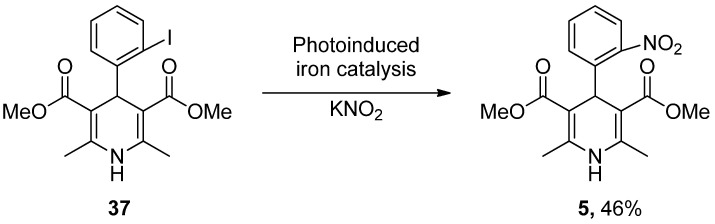
Synthesis of nifedipine **5** via photoinduced iron-catalyzed ipso-nitration.

More recently, nifedipine **5** was synthesized through a photoinduced iron-catalyzed *ipso*-nitration via single-electron transfer [144]. Aryl iodine **37** changed the iodine to nitro substituent to obtain **5** with good yield induced by photocatalysis (Scheme 10).

**Scheme 11:**
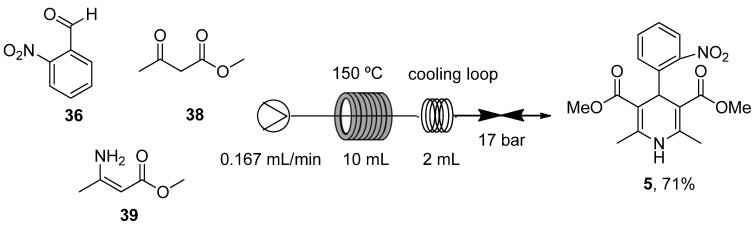
First flow multicomponent synthesis of nifedipine **5**.

The first flow multicomponent production of nifedipine **5** was reported elsewhere [145]. Methanol solutions of compounds **36**, **38**, and **39** were loaded to a heated 10 mL stainless-steel coil reactor (150 °C) at 0.167 mL·min^−1^ to obtain nifedipine **5** in 71% yield (Scheme 11).

**Scheme 12:**
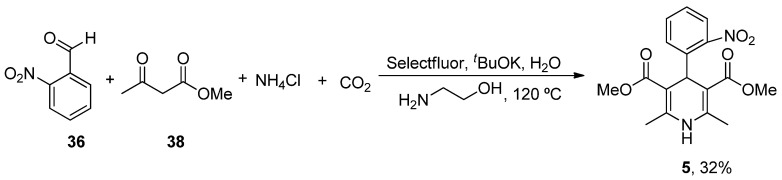
Generation of nifedipine **5** by using the green approach.

A green methodology for nifedipine **5** production was reported [146]. A metal-free multicomponent cycloaddition of ketones with an ammonium cation under a CO_2_ atmosphere was used to obtain dihydropyridine derivatives. Nifedipine **5** was obtained in 32% yield using methyl acetoacetate **38** and o-nitrobenzaldehyde **36** in the presence of ammonium chloride (NH_4_Cl) and CO_2_ in aqueous solution (Scheme 12).

### 6.6. Nimodipine

Nimodipine (Nimotop) is a Ca^2+^ channel blocker utilized to prevent brain damage as a consequence of reduced blood flow. The molecule antagonizes the Cav1.2–1.3 L-type VOCCs, showing a greater affinity for the Cav1.2 channel. As a highly lipophilic compound, nimodipine can easily cross the BBB and reach elevated concentrations in the CSF. The drug has specific binding affinity for dihydropyridine receptors, in contrast to nifedipine that shows predominantly peripheral effects. Nimodipine has been proposed to be of potential therapeutic utility in clinical trials of subjects with AD or PD. The beneficial effects attributable to nimodipine are related to clinical symptoms, cognitive function, and overall physical activity. A multicenter, double-blind, placebo-controlled, randomized clinical trial of nimodipine demonstrated its efficacy to prevent behavioral, cognitive, and affective impairments in patients with primary degenerative dementia of the Alzheimer’s type [147]. A systematic review comprising 14 randomized clinical trials found that treatment with nimodipine improved the Sandoz Clinical Assessment—Geriatric (SCAG) scale and cognitive function on AD-related dementia, as well as mixed AD and cerebrovascular disease [148]. Long-term administration of nimodipine prevented aged-related impaired learning and memory through modulation of synaptosome Ca^2+^-binding proteins [149]. Nimodipine improved reversal spatial learning impairment and synaptic current deficits associated with Aβ pathology [150]. The age-related increase in the L-type Ca^2+^ channel protein α1D in the CA1 region of the hippocampus correlated with higher working memory defect, but nimodipine-treated rats for several months exhibited lower α1D immunoreactivity and improved spatial working memory [151]. Nimodipine ameliorated scopolamine-induced cognitive decline via its regulatory effect on BDNF and acetylcholine [152]. Nimodipine also ameliorated memory failure in aged rhesus monkeys [153]. Exposure to nimodipine reduced Ca^2+^ influx and associated Aβ_1–42_ accumulation [154]. In addition, nimodipine inhibited Aβ_25–35_-mediated toxic effects, including Ca^2+^ uptake and apoptosis. These results suggest that Aβ significantly enhances Ca^2+^ trafficking via nimodipine-sensitive L-type VSCCs, which leads to free radical-induced neuronal loss. Okadaic acid promoted phosphorylation of tau by increasing Ca^2+^ influx through L-type VOCCs, since nimodipine attenuates phospho-tau levels [155]. Tau overexpression increased sensitivity of Ca^2+^ transients to nimodipine [156]. Furthermore, nimodipine limited Aβ aggregation through upregulation of the glutathione S-transferase activity, which results in reduced oxidative damage.

To date, there have been no clinical trials with nimodipine in individuals with PD. Elevated Ca^2+^ content worsened Aβ-mediated behavioral impairment and defective chemotaxis, but exposure to nimodipine extended lifespan and rescued motor lesions, synaptic deficiencies, and DAergic degeneration [157]. Subcutaneous delivery of nimodipine reversed behavioral abnormalities and preserved the number of DAergic neurons in the locus coeruleus [158]. Exposure to nimodipine blocked DA neuron pacemaker activity, which can be restored by virtual Cav1.3 channels [159]. Moreover, nimodipine abolishes autonomous pacemaking in DA neurons and the underlying ΔΨm oscillations [58]. Nimodipine limited MPP^+^-induced increase in cytosolic Ca^2+^ content, mitochondrial depolarization and fragmentation, and neuronal death [160]. In addition, nimodipine also ameliorated MPTP-induced behavioral phenotype and limited striatal DA depletion, SN neuronal loss, and oxidative insult without regulating MAO-B activity. In MPTP-treated mice, treatment with nimodipine protected DA neurons in the SN but no changes were found in the striatum [161]. Nimodipine had no effect on behavioral impairment and striatal DA depletion but preserved DA neurons from death in marmosets injected with MPTP [162]. Nimodipine simultaneously upregulated DA release whilst suppressing AChE release. Nimodipine prevented dendritic spine loss and behavioral abnormalities but not associated rotational asymmetry in DA-grafted rats [163]. Exposure to nimodipine did not impact DA graft survival but promoted graft reinnervation of striatum. Continuous-release pellets of nimodipine prevented locomotor disturbances in unilateral 6-OHDA mesencephalic lesions [164]. Nimodipine significantly decreased the amount of NO^•^ and LPS-activated microglia [165]. Nimodipine also downregulated DA uptake in neuron–glia cultures from mice lacking functional NADPH oxidase (involved in the production of O2^•−^). In summary, the L-type Ca^2+^ channel blocker antagonist nimodipine shows beneficial effects on AD and PD. The molecule ameliorates behavioral outcome, increases synaptic transmission and neuron survival, improves mitochondrial function, and attenuates oxidative stress and inflammation. Nimodipine is, in general, well tolerated although sensations of warmth and skin reddening can occur. High concentrations of the nimodipine can result in reduced blood pressure, headache, nausea, muscle weakness, and gastrointestinal issues [147]. Isolated CNS symptoms, such as insomnia, tachycardia, and increased motor activity have also been reported.

**Scheme 13:**
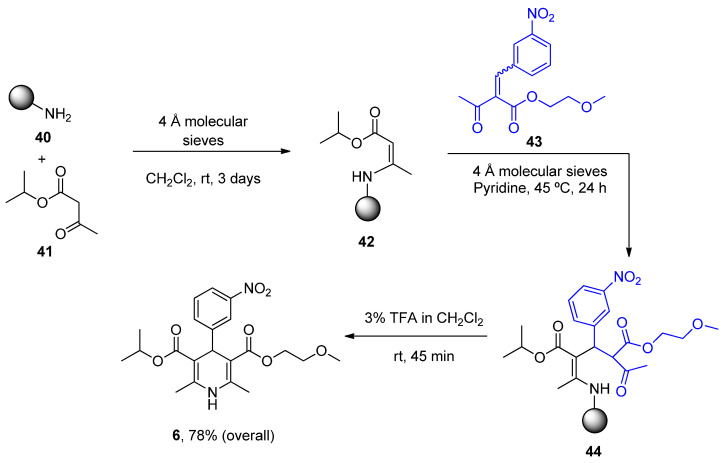
Solid-phase production of nimodipine **6**.

A solid-phase synthesis was reported by Gordeev et al. for the preparation of nimodipine (3-(2-Methoxyethyl) 5-propan-2-yl 2,6-dimethyl-4-(3-nitrophenyl)-1,4-dihydropyridine-3,5-dicarboxylate) **6** and other bioactive dihydropyridines [166]. Condensation of the immobilized *N*-tethered enamino component **42** with the corresponding 2-benzylidene α-keto ester **43** provided the conjugated enamine **44**. This intermediate was treated with trifluoroacetic acid to obtain the free enamino ester, which spontaneously cyclizes in solution to obtain the desired product **6** (Scheme 13).

**Scheme 14:**
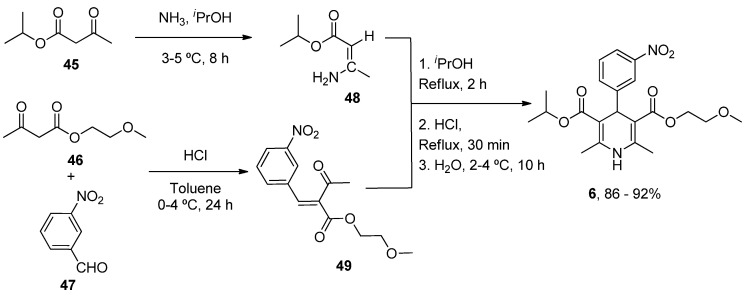
Production of nimodipine **5** in pilot batches by Pharm. Sintez. Co (Moscow, Russian Federation).

More recently, due to a considerable demand of nimodipine in the Russian market, Pharm. Sintez Co. developed a new methodology to produce the drug in pilot batches [167]. The approach includes the production of 1-methylethyl-3-amino-crotonate **48** and 2-methoxyethyl-2-(3-nitrobenzyl-idene)acetoacetate **49**. Cyclocondensation of both compounds employing *i*PrOH as the solvent, in the presence of hydrochloric acid, afforded the final product **6** in high yield and purity (Scheme 14).

## 7. Conclusions and Future Directions

Multiple candidate drugs for treating AD or PD have failed over the last years. Further research is still needed to determine the therapeutic time window and the stage of the disease that should be used in clinical trials. Neuropathological and biochemical assessments support the notion that mitochondrial dysfunction causes an increase in free radicals and Ca^2+^-induced toxicity, which are important factors in the neurodegenerative process described in AD and PD. According to the combination of preclinical and clinical data, there is a general consensus that development of therapeutic interventions targeting mitochondrial Ca^2+^ signaling may slow or stop the progression of neurodegenerative diseases, including AD and PD. A set of strategies to normalize mitochondrial Ca^2+^ homeostasis and signaling has been described. One potential approach involves Ca^2+^ uptake blockade through the L-type VOCCs, which inhibits Ca^2+^ transient at either presynaptic or postsynaptic sites and attenuates glutamate-mediated excitotoxicity. Selective antagonists of Cav1.2–1.3 L-type VOCCs also exhibit beneficial properties since prolonged opening of L-type channels results in augmented Ca^2+^ release and influx, as well as its accumulation in DA neurons. The capability of heterocyclic agents to modulate mitochondrial Ca^2+^ content and signaling, and their ability to preserve mitochondrial biogenesis and function provide a plausible biological basis for their neuroprotective effect (Table 1).

## Figures and Tables

**Table 1:** Summary of heterocycle compounds effects on AD and PD.

Heterocyclic Compound	Chemical Name	Target	Proposed Mechanism	Effect on Neurodeg. Dis. Clinical Trial Status	References (Clinical Trials)
ANAVEX2-73 (Blarcamesine)	Tetrahydro-*N*,*N*-dimethyl-2,2-diphenyl-3-furanmethanamine hydrochloride	mAChRs S1R	↓ Ca^2+^ release ↓ Mitochondrial stress Activated antioxidant response pathways Limited apoptosis	↓ Risk of developing AD and PD AD (phase IIb/3) AD (phase III) PDD (phase II) PDD (phase III)	[62] ANAVEX2-73-AD-004 NCT03790709 NCT04575259 NCT03774459
Caffeine (mateine)	1,3,7-Trimethyl-3,7-dihydro-1*H*-purine-2,6-dione	A2AR RyR (+)	↓ Oxidative damage ↓ Aβ levels ↓ α-Syn aggregates Restored AChE and Na^+^/K^+^ ATPase activity	↓ Risk of developing AD and PD Epidemiological studies: motor benefits in PD	[73,74,75] [81,82,83,84,85,86,87,88]
Diltiazen (Cardizem)	(2*S*,3*S*)-5-(2-(Dimethylamino)ethyl)-2-(4-methoxyphenyl)-4-oxo-2,3,4,5-tetrahydrobenzo[*b*][1,4]thiazepin-3-yl acetate	Non-dihydro-pyridine VOCC	↓ Ca^2+^ entry ↓ Oxidative damage ↓ Inflammation	↓ Risk of developing PD Epidemiological and randomization studies	[102]
Latrepirdine (Dimebon)	3,6-Dimethyl-9-(2-methyl-pyridyl-5)-ethyl-1,2,3,4-tetrahydro-γ-carboline dihydrochloride	H1R Other Ca2^+^ Channels	↓ Ca^2+^ release and entry ↓ Aβ levels ↓ Oxidative damage to lipids Inhibited AChE ↓ α-Syn	Cognitive and psychiatric benefits in AD Phase III AD: discontinued	[112,113,114] NCT00838110
Nifedipine (Procardia)	3,5-Dimethyl 2,6-dimethyl-4-(2-nitrophenyl)-1,4-dihydropyridine-3,5-dicarboxylate	L-Type VOCC	↓ Ca^2+^ entry ↓ Aβ production ↓ Oxidative damage	Does not ↓ risk of developing PD Epidemiological and randomization studies	[134]
Nimodipine (Nimotop)	3-Isopropyl 5-(2-methoxyethyl) 2,6-dimethyl-4-(3-nitrophenyl)-1,4-dihydropyridine-3,5-dicarboxylate	L-Type VOCC	↓ Ca^2+^ entry Neuroprotection ↓ Aβ toxicity ↓ Oxidative damage Abolished pacemaking activity in DA neurons	↓ Risk of developing AD Epidemiological and randomization studies	[147,148]

## Abbreviations

Ac: acetate; DBU: 1,8-diazabicyclo[5.4.0]undec-7-ene; DMAP: 4-dimethylaminopyridine; DMF: dimethylformamide; DMSO: dimethyl sulfoxide; Dppf: 1,1′-*bis*(diphenylphosphino)ferrocene; GDH: glutamate dehydrogenase; NADP^+^: nicotinamide-adenine dinucleotide phosphate; Tf: trifluoromethanesulfonate; TFA: trifluoroacetic acid; THF: tetrahydrofuran; Tos: Ts: toluenesulfonyl.
